# HLA-DR expression in neonates after cardiac surgery under cardiopulmonary bypass: a pilot study

**DOI:** 10.1186/s40635-017-0166-x

**Published:** 2018-01-11

**Authors:** Alexis Chenouard, Cécile Braudeau, Nicolas Cottron, Pierre Bourgoin, Nina Salabert, Antoine Roquilly, Régis Josien, Nicolas Joram, Karim Asehnoune

**Affiliations:** 1 CHU Nantes, Service de Réanimation Pédiatrique, Nantes, France; 2 CHU Nantes, Laboratoire d’Immunologie, CIMNA, Nantes, France; 3 Centre de Recherche en Transplantation et Immunologie, UMR 1064, INSERM, Université de Nantes, Nantes, France; 4 LabEx IGO “Immunotherapy, Graft, Oncology”, Nantes, France; 5 CHU Nantes, Pôle anesthésie réanimations, Service d’anesthésie réanimation chirurgicale, Hôtel Dieu, Nantes, France

## Abstract

Monocyte HLA-DR expression has been reported as a marker of immunosuppression and a predictor of sepsis development. However, to date, there is no report on monocyte HLA-DR monitoring exclusively in neonates (< 28 days of life) who underwent cardiac surgery under cardiopulmonary bypass (CPB), which have a high risk of nosocomial infection. In this pilot study, we studied nine neonates with a diagnosis of congenital heart disease requiring surgery under CPB. There was a significant reduction in monocyte HLA-DR expression for the first two postoperative days, as compared to preoperatively (*p* = 0.004). Moreover, neonates who displayed an episode of NI had a dramatically lower HLA-DR expression at day 4, as compared to neonates without NI (4257 AB/c [2220–5895] vs 14,947 AB/c [9858–16,960]; *p* = 0.04). Our preliminary results could indicate that HLA-DR expression may be a useful biomarker of immunosuppression-induced secondary infection after CPB in neonates.

## Background

The pro-inflammatory response that accompanies the onset of critical illness often occurs concurrently with a compensatory anti-inflammatory response. When severe and persistent, this anti-inflammatory response has been termed immunoparalysis. The diminished monocyte human leukocyte antigen-DR (HLA-DR) expression on cell surface is proposed to reflect immunoparalysis in critically ill patients [1, 2]. To date, HLA-DR expression has been assessed in adults as a predictor of septic complications after various injuries [3–6]. In pediatric cardiac surgery, HLA-DR expression has been examined in two studies [7, 8]. However, the results were principally limited by concern related to age of patient with a large heterogeneity [8] or non-inclusion of children younger than 3 months [7]. To our knowledge, no study has specifically focused on HLA-DR expression on circulating monocytes among neonates who underwent cardiac surgery under cardiopulmonary bypass (CPB), which have a high risk of nosocomial infection (NI) [9]. In this pilot study, we investigated the kinetic of monocyte HLA-DR expression in this population and described the relationship between monocyte HLA-DR expression and the subsequent development of NI.

## Methods

Blood samples were collected from neonates preoperatively at line insertion, and 1, 2, 3, and 4 days after the end of CPB on immunology laboratory working days (Monday to Friday). The number of HLA-DR molecules per monocyte (AB/c) was determined immediately after sample collection (i.e., within 90 min) by flow cytometry on whole blood using a standardized method with a Quantibrite phycoerythrin fluorescence quantitation kit (Quantibrite anti HLA-DR/Anti Monocytes CD14, BD Biosciences, Le Pont de Claix, France), as previously described [10, 11]. Blood samples for cytokine assays were immediately centrifuged, and the plasma was stored at − 80 °C. Cytokine concentrations in the plasma (IL-6, IL-8, and IL-10) were measured by multiplex immunoassay according to the manufacturer’s protocol (Merck Millipore, Molsheim, France). Immunological analysis was conducted blind, and clinical data were not available to the immunology staff before the end of the study.

NI including catheter-related bloodstream infections, ventilator-associated pneumonia, and sternal wound infections were defined based on the Center of Disease Control (CDC) and National Nosocomial Infections Surveillance criteria [12] and were prospectively recorded during the PICU stay or within 30 days after surgery. All parents were informed of the project and written consent was waived. The study was approved by the local ethics committee of the University Hospital of Nantes.

### Statistics

Statistical analyses were performed using GraphPad Prism software (GraphPad, La Jolla, CA). The Kruskal–Wallis test was used for comparisons of multiple groups (preoperatively, 1, 2, 3, and 4 days after CPB). Dunn’s multiple comparisons test was used as a post hoc test for intergroup comparisons. Continuous nonparametric variables were expressed as medians (extremes values). The Mann–Whitney test was used to compare two independent groups on day 4 (infected versus non-infected patients). Significance was defined as *p*-value less than 0.05.

## Results

**Fig. 1:**
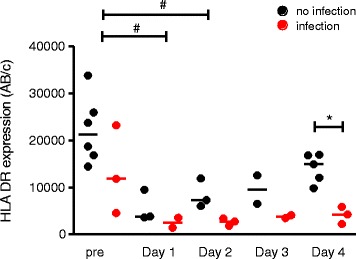
HLA-DR expression in neonates after cardiopulmonary bypass. Each point in red represents expression of HLA-DR in patients who displayed an episode of NI. The Kruskal–Wallis test was used for comparisons of multiple groups, with Dunn’s multiple comparisons test for intergroup comparisons (preoperatively, 1, 2, 3 and 4 days after CPB) (# *p* < 0.05). The Mann–Whitney test was used to compare infected versus non-infected patients on day 4 (*p < 0.05)

Table 1 depicts the baseline characteristics of the nine neonates included in this study that underwent congenital cardiac surgery under CPB. As shown in Fig. 1, there was a significant reduction in monocyte HLA-DR expression for the first two postoperative days, as compared to preoperatively (*p* = 0.004). Among the three patients (33%) who displayed an episode of NI (two sternal wound infections and one ventilator-associated pneumonia respectively on days 3, 4, and 8 after CPB), we observed a dramatic lower HLA-DR expression at day 4, as compared to patients without NI (4257 AB/c [2220–5895] vs 14,947 AB/c [9858–16,960]; *p* = 0.04) (Fig. 1). Of note, among infected patients, two present low HLA-DR values before surgery. As lower gestational age and birth weight was known to be associated with diminished HLA-DR expression [13], we analyzed the preoperatively HLA-DR expression according to the gestational age and the birth weight of patients, and we did not find any correlation in our cohort (data not shown). Finally, ratio of HLA-DR expression between day 4 and days 1–2 (minimal value between day 1 and day 2) was 1.5 [1.2–2.1] in the infected group compared to 2.5 [1.4–3.2] in the non-infected group (*p* = 0.23).

**Table 1:** Characteristics of patients (*n* = 9)

Demographics	
Age (days)	12 [6–23]
Weight (kg)	2.9 [2.0–4.0]
Male, *n* (%)	7 (78%)
Gestational age < 36 weeks, *n* (%)	2 (22%)
Genetic abnormality, *n* (%)	0 (0%)
Cyanotic congenital heart defects, *n* (%)	5 (56%)
Lymphocyte cell count preoperatively (/mm^3^)	5350 [2000–7510]
Characteristics of surgery	
RACHS-1 score ^a^	4 [3–6]
Time on cardiopulmonary bypass (min)	164 [54–272]
Aortic cross-clamp time (min)	85 [0–182]
Hypothermia (20–28 °C), *n* (%)	5 (56%)
Characteristics during the PICU stay after surgery	
Use of corticoids during the first 48 h, *n* (%)	6 (67%)
Extracorporeal membrane oxygenation support, *n* (%)	1 (11%)
Delayed closure of sternum, *n* (%)	4 (44%)
Peak Vasoactive-Inotropic score during the first 48 h ^b^	11 [2–56]
Positive fluid balance at day 2, *n* (%)	2 (22%)
Acute kidney injury at day 2 ^c^, *n* (%)	4 (44%)
Lymphocyte cell count at day 2 (/mm^3^)	1830 [1100–4000]
Time on mechanical ventilation (days)	4 [2–10]
Nosocomial infection, *n* (%)	3 (33%)
PICU length of stay (days)	8 [4–80]
Death, *n* (%)	1 (11%)

Data are expressed as medians (extremes values) or *n* (%)

^a^RACHS-1 (Risk-Adjusted classification for Congenital Heart Surgery) score reflects procedure complexity (range, 1–6), with higher scores correlating with more complex surgery [15]

^b^Vasoactive-Inotropic score = (1 × dopamine [μg/kg/min] + 1 × dobutamine [μg/kg/min] + 100 × epinephrine [μg/kg/min] + 100 × norepinephrine [μg/kg/min] + 10 × milrinone [μg/kg/min] + 10,000 × vasopressin [U/kg/min]) [16]

^c^Acute kidney injury is defined by using the pediatric-modified RIFLE criteria (“Failure” category) [17]

**Fig. 2:**
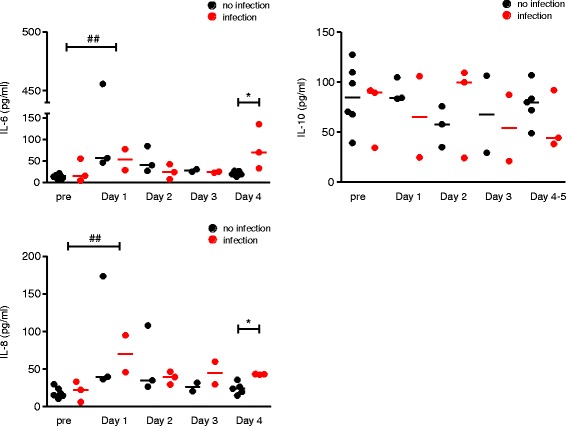
Circulating levels of IL-6, IL-8 and IL-10 in neonates after cardiopulmonary bypass. The Kruskal–Wallis test was used for comparisons of multiple groups, with Dunn’s multiple comparisons test for intergroup comparisons (preoperatively, 1, 2, 3 and 4 days after CPB) (## *p* < 0.01). The Mann–Whitney test was used to compare the infected versus non-infected patients on day 4 (*p < 0.05)

We next evaluated the inflammatory cytokine response after CPB. As shown in Fig. 2, circulating levels of pro-inflammatory cytokines IL-6 and IL-8 were significantly higher on day 1 as compared to preoperatively (*p* < 0.01), whereas the release of anti-inflammatory IL-10 cytokine was not affect by CPB. At day 4, patients with NI showed increased IL-6 and IL-8 release as compared to patients without NI (70 pg/ml [33–135] vs 19 pg/ml [14–27] and 43 pg/ml [42–43] vs 24 pg/ml [15–36], respectively; *p* = 0.04) (Fig. 2).

## Discussion

In this pilot study, we report that neonates had a dramatic reduction in HLA-DR expression on circulating monocytes during the first two postoperative days after CPB, and those with prolonged decreased HLA-DR in the early postoperative period (day 4) could represent a subpopulation at greatly increased risk of later NI. Moreover, immunoparalysis described here after neonatal CPB accompanies a pro-inflammatory response, illustrated by high circulating levels of IL-6 and IL-8. This increase in pro-inflammatory cytokines IL-6 and IL-8 24 h after CPB is consistent with previous studies in pediatric cardiac surgery [14].

The main limitation of this preliminary study concerns the fact that HLA-DR values were not censored after NI diagnosis given the small number of patients included. These promising findings warrant thus a larger confirmatory trial (NCT03309839) before HLA-DR expression can be introduced in the clinical practice as a useful biomarker of immunosuppression-induced after CPB in neonates.
